# A prospective study to evaluate the accuracy of pulse power analysis to monitor cardiac output in critically ill patients

**DOI:** 10.1186/1471-2253-8-3

**Published:** 2008-02-18

**Authors:** Maurizio Cecconi, Jayne Fawcett, R Michael Grounds, Andrew Rhodes

**Affiliations:** 1 Department of Intensive Care Medicine, St George's Hospital, London, SW17 0QT, UK

## Abstract

**Background:**

Intermittent measurement of cardiac output may be performed using a lithium dilution technique (LiDCO). This can then be used to calibrate a pulse power algorithm of the arterial waveform which provides a continuous estimate of this variable. The purpose of this study was to examine the duration of accuracy of the pulse power algorithm in critically ill patients with respect to time when compared to measurements of cardiac output by an independent technique.

**Methods:**

Pulse power analysis was performed on critically ill patients using a proprietary commercial monitor (PulseCO). All measurements were made using an in-dwelling radial artery line and according to manufacturers instructions. Intermittent measurements of cardiac output were made with LiDCO in order to validate the pulse power measurements. These were made at baseline and then following 1, 2, 4 and 8 hours. The LiDCO measurement was considered the reference for comparison in this study. The two methods of measuring cardiac output were then compared by linear regression and a Bland Altman analysis. An error rate for the limits of agreement (LOA) between the two techniques of less than 30% was defined as being acceptable for this study.

**Results:**

14 critically ill medical and surgical patients were enrolled over a three month period. At baseline patients showed a wide range of cardiac output (median 7.5 L/min, IQR 5.1 -9.0 L/min). The bias and limits of agreement between the two techniques was deemed acceptable for the first four hours of the study with percentage errors being 29%, 22%, and 285 respectively. The percentage error at eight hours following calibration increased to 36%. The ability of the PulseCo to detect changes in cardiac output was assessed with a similar analysis. The PulseCO tracked the changes in cardiac output with adequate accuracy for the first four hours with percentage errors being 20%, 24% and 25%. However at eight hours the error had increased to 43%.

**Conclusion:**

The agreement between lithium dilution cardiac output and the pulse power algorithm in the PulseCO monitor remains acceptable for up to four hours in critically ill patients.

## Background

The measurement of cardiac output is an important component of the management of critically ill haemodynamically unstable patients[1,2]. In recent years there has been increasing emphasis towards the less invasive or minimally- invasive monitoring tools [3-10]. There are a number of commercially available monitors that measure cardiac output from an intra-arterial pressure line. These utilize different algorithms to relate changes in arterial pressure to changes in stroke volume and thus cardiac output [1,2,4,8,9,11]. To date the most accepted of these devices have required an independent form of validation or calibration of cardiac output. This calibration remains valid so long as there are no significant changes in the haemodynamic status of the patients. It is unclear as to when and under what conditions this re-calibration should be performed.

The LiDCOplus monitor (LiDCO, Cambridge, UK) is a device that combines a pulse power algorithm (PulseCO) with an independent form of calibrating the pulse power algorithm via lithium dilution (LiDCO) [3]. This device has been validated in a number of different clinical and veterinary conditions. To our knowledge there are no data showing how well the algorithm maintains its accuracy during an eight hour interval free of calibration in a mixed (medical/surgical) adult intensive care population of critically ill patients. This study, therefore seeks to investigate the duration that the two methods remain sufficiently similar to be acceptable for clinical use.

## Methods

### Patients

Adult critically ill patients who for clinical reasons were being monitored with the LiDCOplus cardiac output monitor on the General Intensive Care Unit at St George's Hospital were enrolled into the study. All patients, or their next of kin where appropriate, gave written informed consent to the study as had been previously agreed with the Local Research Ethics Committee of St George's Healthcare NHS Trust. All patients were critically ill and had both a radial arterial catheter and a central venous line in-situ. Any patient who was less than 40 kg in weight or had significant aortic regurgitation was excluded from this study.

### Protocol

At baseline the PulseCO was calibrated using the lithium dilution technique as previously described and according to manufacturer's instructions[8]. This method involves the placing of 0.3 mmols of lithium chloride in an indwelling central venous line and then the rapid flushing of this line with 20 ml of 0.9% saline. This ensures a rapid bolus of lithium chloride is introduced into the circulation. A lithium specific sensor connected to the arterial line then detects the change in lithium ion concentration in blood that is then passed across the sensor at a fixed rate by a specialized pump. The detection of this concentration by the sensor generates a lithium dilution curve. This is analysed and a reference cardiac output generated. This is the procedure that is recommended by the manufacturers of the device.

A LiDCO measurement was taken at baseline and then at 1, 2, 4 and 8 hours subsequently. These were done only during intervals relatively free of haemodynamic change. This was defined as being five minutes in which there were no alterations in vasoactive medications, no contemporary fluid challenges, no changes in heart rate (HR) or changes in mean arterial pressure (MAP) of more than 10 %. The PulseCO measurement was equalized to the LiDCO measurement only at baseline. Subsequent measurements of PulseCO at the same intervals as the measurements of the LiDCO were taken but the measurements were not equalized at these points to reset the PulseCO system. The PulseCO value was recorded as the mean of the value immediately before and after the lithium dilution.

### Statistical analysis

Data are expressed as means with a standard error when normally distributed and a median with an interquartile range when not. In this study the measurement of cardiac output from lithium dilution was considered the reference method for comparison. PulseCO measurements were thus compared against the LiDCO measurement for each individual time-point. This study is comparing two independent methods of measuring cardiac output each with their own inherent rate of error. Comparison between these measurements was performed by linear regression analysis and the technique described by Bland and Altman[12]. We defined a level of agreement between the two techniques of less than 30% as being clinically acceptable as described by Critchley and Critchley[13]. This value is based on an assumption that for cardiac output monitoring a new device should have a similar level of precision to the intermittent thermodilution from a pulmonary artery catheter that has a precision of approximately 10%. If the new tested device was to have a similar precision, then the percentage error for agreement between the two techniques should be less than 28% as calculated by dividing twice the standard deviation of the differences by the mean cardiac output for both techniques. The PulseCO algorithm is designed to track changes in cardiac output. We therefore also analysed the data with respect to changes in this variable. The absolute values of these changes are not as important as the percentage change in relation to the actual value, so this is presented as a percentage change from the previous reading and plotted as both a linear regression plot and a Bland Altman analysis [14]. Single plots of PulseCo and LiDCO against time for each patient were made in order to show the agreement between the two measurements for individual patients. All analyses were performed using Graphpad Prism software system of Graphpad Software Inc.

## Results

Fourteen patients were enrolled into the study. Six of these patients were male and eight female. They had a median age of 67.5 (29–76) years, a median height of 1.66 (1.6–1.73) m and a median weight of 67 (58–102) kg. Five of the patients were post surgery and all were critically ill from a number of different aetiologies [Table 1]. Eight patients had multiple organ failure due to sepsis (6 medical, 2 surgical). The remaining six patients had acute heart failure (3 medical, 2 post partum, 1 surgical). In all fourteen patients measurements were available at baseline, 1 hour, 2 hours and 4 hours. In two patients data at eight hours were unavailable due to clinical priorities meaning the patients had had to be removed from the study [Table 2]. All the measurements were suitable for analysis. A total number of 54 pairs of data were available at the end of data collection.

**Table 1:** Patient Characteristics at Baseline. HAP is hospital acquired pneumonia, CAP is community acquired pneumonia.

**Patient**	**Age**	**Sex**	**Height (cm)**	**Weight (kg)**	**Diagnosis**
1	53	Male	190	80	Acute pancreatitis
2	32	Female	159	150	Acute heart failure post pregnancy
3	68	Female	166	45	Septic shock secondary to CAP
4	65	Female	160	110	Septic shock secondary to abdominal sepsis
5	28	Female	160	53	Acute heart failure post pregnancy
6	33	Male	184	106	Septic shock secondary to abdominal sepsis
7	89	Female	160	50	Haemorrhagic shock
8	62	Male	165	57	Septic shock secondary to CAP
9	84	Female	174	60	Acute heart failure following haemorrhagic shock
10	72	Male	178	189	Septic shock secondary to HAP
11	83	Male	170	60	Septic shock secondary to HAP
12	71	Female	148	70	Septic shock secondary to HAP
13	76	Male	169	90	Septic shock
14	73	Female	152	65	Cardiogenic shock

**Table 2:** Patient haemodynamics at baseline and after eight hours of study or at study completion.

	**Baseline Measurements**				**Measurements at completion of study**			
	**HR**	**MAP**	**CO**	**NorEpi**	**HR**	**MAP**	**CO**	**NorEpi**

1	103	96	8.03	0	93	95	6.04	0
2	85	119	9.01	0	97	130	9.06	0
3	101	69	4.05	0.02	106	77	6.02	0.02
4*	116	82	10.03	0.06	103	80	10.03	0.06
5	129	118	8.02	0	128	113	7.07	0
6*	121	80	14.08	0.01	118	85	15.01	0.01
7	68	85	2.08	0	90	78	4.01	0
8	118	79	8.08	0.02	71	99	5.06	0.02
9	82	100	3.07	0	110	100	3.06	0
10	117	66	18.03	0.02	109	70	14.05	0.02
11	115	80	4.09	0	134	71	5.03	0
12	99	66	6.02	0	108	76	5.00	0
13	105	64	5.06	0.03	110	72	4.07	0.03
14	89	83	6.08	0.01	83	84	5.02	0.01

HR is heart rate (/minute), MAP is mean arterial pressure (mmHg), CO is cardiac output (L/min) and NorEpi is dose of norepinephrine in mcg/kg/min. * describes the patients where the second set of measurements was taken at four hours rather than eight due to being removed from the study.

The baseline data show a large range of cardiac outputs (LiDCO from 2.8 to 18.3 L/min) with a median cardiac output of 7.5 (5.1 to 9.0) L/min. The median heart rate was 104 (92–117) beats per minute, median arterial pressure 81 (72–93) mmHg and median central venous pressure 16 (12–19) mmHg [Table 2]. Seven patients were receiving a norepinephrine infusion to support the mean arterial pressure at the beginning and throughout the study period. Over the eight hour study period six of the fourteen patients had changes in their cardiac output of greater than 15% from their baseline value [Table 2].

**Figure 1:**
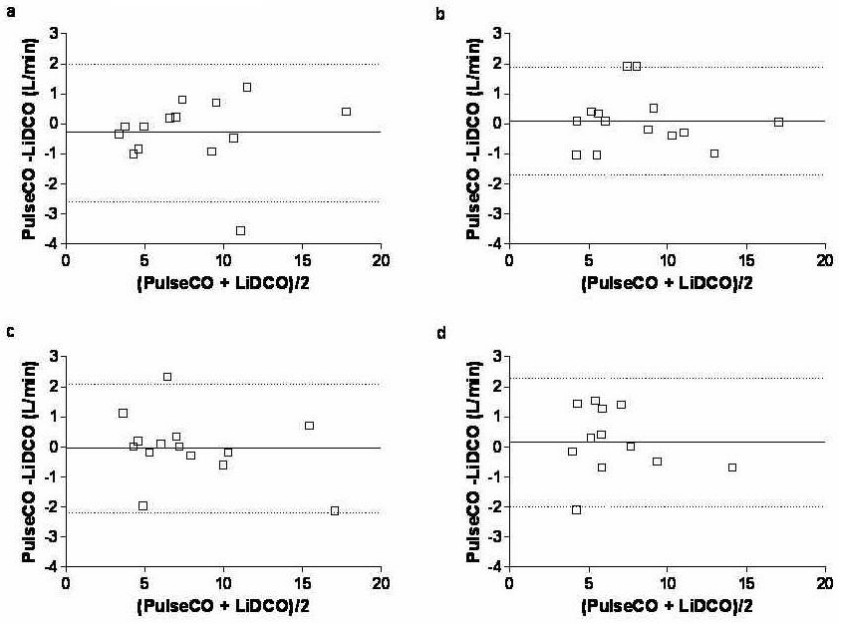
Linear regression and Bland Altman plots for PulseCO versus LiDCO at 1 (a), 2 (b), 4 (c) and 8 (d) hours following calibration. The solid lines in the Bland Altman plot represent the bias and the dotted lines represent the limits of agreement (2 × standard deviation of the bias).

At baseline, the cardiac output of the PulseCO system was equalized with the independent form of measurement (the LiDCO) meaning that the two monitors displayed exactly the same number. This absolute value of cardiac output from the PulseCO system remained acceptable as compared to the LiDCO for the next four hours of the study. Data for PulseCO and LiDCO at 1, 2 and 4 hours demonstrated an acceptable levels of bias and limits of agreement for the first four hours of the study, however at eight hours following calibration the PulseCO device had a percentage error that was outside of the acceptable range [Figure 1]. After one hour of study, the bias and limits of agreement between PulseCO and LiDCO were -0.3 ± 2.3 L/min with a percentage error of 29%, at two hours 0.1 ± 1.9 (22%), at four hours -0.1 ± 2.2 (28%) and at eight hours 0.2 ± 2.2 (36%) [Table 3].

**Table 3:** Table demonstrating haemodynamic measurements from the LiDCO and PulseCO monitors for up to eight hours. HR, MAP, CVP, LiDCO and PulseCO (median and IQR), bias and percentage error at measurements intervals.

	Baseline	+1 hour	+ 2 hours	+4 hours	+8 hours
HR/min	104 (92–117)	110 (96–123)	111 (95–117)	107 (94–117)	107 (92–110)
MAP mmHg	81 (72–93)	84 (77–93)	86 (75–98)	81 (75–88)	81 (75–99)
CVP mmHg	16 (12–19)	16 (10–20)	13 (11–18)	16 (11–21)	15 (11–16)
LiDCO L/min	7.5 (5.1–9.0)	7.0 (5.0–10.6)	6.8 (5.6–10.1)	6.4 (5.3–9.8)	5.5 (4.9–6.7)
PulseCO L/min	#	7.5 (4.4–9.8)	8.6 (5.5–9.9)	7.2 (4.8–9.2)	6.1 (5.2–7.7)
Bias ± 2SD L/min	#	-0.3 ± 2.3	0.1 ± 1.9	-0.1 ± 2.2	0.2 ± 2.3
Percentage error	#	29%	22%	28%	36%

**Figure 2:**
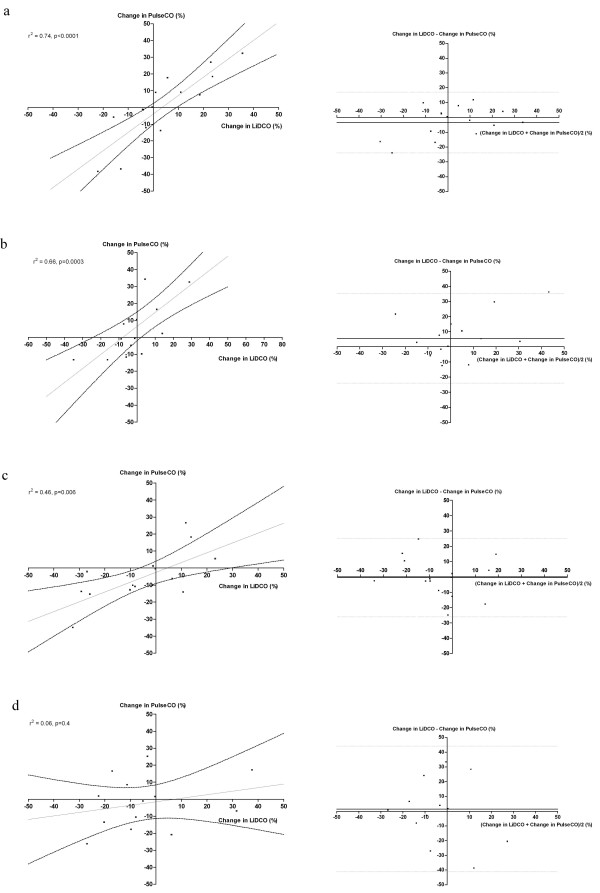
Linear regression and Bland Altman plots for percentage changes in PulseCO versus changes in LiDCO at 1 (a), 2 (b), 4 (c) and 8 (d) hours following calibration. The solid line in the regression plots represents the regression line, the dotted lines represent the 95% confidence intervals around this line. The solid lines in the Bland Altman plot represent the bias and the dotted lines represent the limits of agreement (2 × standard deviation of the bias).

The PulseCO system utilizes a pulse power algorithm to track changes in cardiac output from a baseline value. It is therefore important to also assess whether the changes in cardiac output between the two devices were of similar direction and magnitude. In this study there were significant agreements between the two techniques of detecting changes in cardiac output for the first four hours following calibration. This is evidenced from the significant correlation between percentage changes in the cardiac output from the preceding time point as measured by the PulseCO when compared to changes as measured by the LiDCO method (r^2 ^0.46–0.76, p < 0.006). At eight hours the changes in cardiac output were not significantly correlated between the two devices [Figure 2]. The bias and limits of agreement for the percentage changes in the cardiac outputs between the two measurements were as follows: at one hour -3.5 ± 20%, at two hours 5.6 ± 24%, at four hours -0.4 ± 25% and at eight hours 1.4 ± 43% [Figure 2].

**Figure 3:**
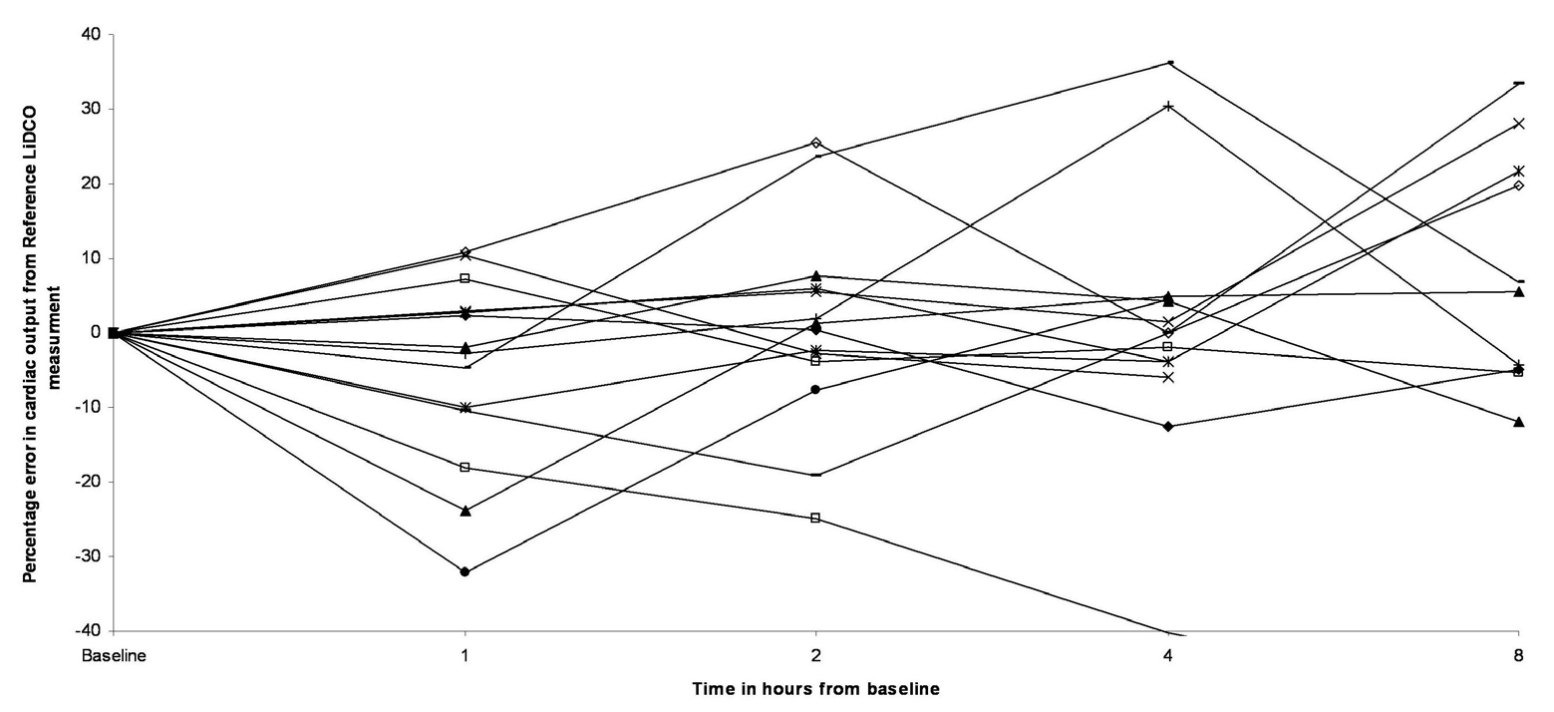
Percentage errors between PulseCO and LiDCO for Individual patients over an eight hour period.

**Figure 4:**
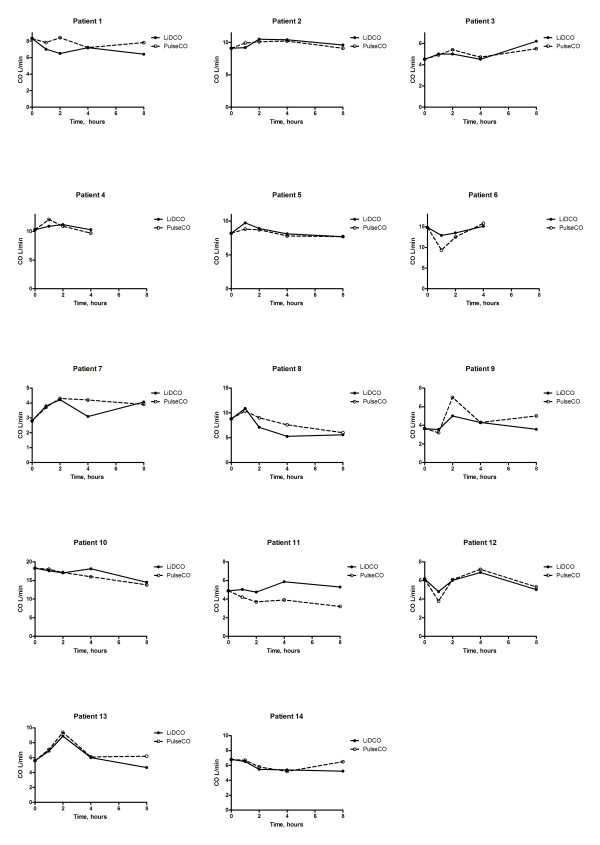
Graphs representing values of cardiac output for each individual patient as measured by both LiDCO and PulseCO.

The separate plots of PulseCo and LiDCO against time for each patient [Figure 4] show how the two techniques track cardiac output. The graphs show that for the majority of patients the magnitude and direction of change in cardiac output is similar indicating that the two devices track cardiac output appropriately. In a few patients, this relationship is not so good, and this partly explains the percentage error for the limits of agreement that are detailed above. Although the accuracy of cardiac output measured by the PulseCO system remained adequate for the first four hours of the study it is important to recognize that in individual patients the accuracy over time varied considerably [Figure 3] with a tendency for the percentage errors to increase [Figure 3]. The six patients with the highest changes in cardiac output, however, did not have the largest percentage errors in the readings of cardiac output between LiDCO and PulseCO, with the average error in this group being 17%.

## Discussion

This study assesses how well the PulseCO algorithm maintains its accuracy in a mixed group of critically ill patients. We deliberately chose to study critically ill patients whose cardiac output was being monitored for clinical reasons, as these are often the groups of patients that these devices are used in, but are rarely the groups of patients that they are validated for. Despite these challenging conditions we were able to demonstrate that the PulseCO algorithm is an acceptable method to measure cardiac output for up to four hours without re-calibration.

Continuous measurement of cardiac output is becoming increasingly popular. The two most popular devices that provide continuous CO from the analysis of arterial pressure waveform are the PiCCOplus (Pulsion medical system, Munich, Germany) [1,4,5]and Lidco™plus (LidCO, Cambridge, UK) [3,7-10]. PiCCOplus has been commercially available for longer and both its calibration system and continuous cardiac output system have been validated in different clinical scenes. The algorithm has been validated against both pulmonary and transpulmonary thermodilution. It has been demonstrated to be accurate so long as re-calibration is performed in case of major haemodynamic change[1,2,5]. Most of the validation studies of continuous cardiac output monitoring from the PiCCO system have been performed in patients undergoing either cardiac surgery or post operative patients on the Intensive Care Unit. Less is known about the accuracy of these continuous CO monitors in the critically ill population of patients.

LiDCO™plus, a more recently available system. has been validated against the pulmonary artery catheter and trans-pulmonary thermodilution in animals[3,6,11] and in different clinical scenarios in humans [7] including cardiac surgery and in post surgical patients [8-10]. There are no data comparing subsequent readings from the PulseCO (after initial calibration) in a mixed adult intensive care population. Pitmann et al, compared PulseCO and LiDCO in post surgical patients[8]. Their data showed a good bias and limits of agreement at four and eight hours with an overall error of 27%. A previous study by Hamilton et al had also shown good agreement between PulseCO and LiDCO in patients following cardiac surgery [9]. In this study a similar protocol to ours was used, calibrating PulseCO at the beginning and then measuring PulseCO, LiDCO and intermittent thermodilution CO from the pulmonary artery catheter at baseline, 2, 4, 6 and 8 hours. Good agreements were found between the three methods. The authors concluded that following cardiac surgery PulseCO can be used without recalibration for at least eight hours.

This study has assessed the ability of the pulse power algorithm to maintain its accuracy when compared to the LiDCO against time. It has not compared the accuracy of the combination of LIDCO and PulseCO to give the absolute cardiac output. This would need a further independent measurement technique of cardiac output as a reference. In our population we found an acceptable agreement between PulseCO and LiDCO for the first four hours following calibration of the device and a reasonable ability of the algorithm to track changes in this variable within this time window. This agreement may in part relate to the wide range of cardiac outputs that our patients presented with in comparison to the relatively small changes in the variable seen over the first few hours. This raises the possibility that the relationship may in fact be a spurious artefact of the analysis rather than a real phenomenon. Four hours following calibration, however, the percentage error increased to levels that we had pre-defined as not being adequate. We calculated the percentage error of the limits of agreement as described by Critchley and Critchley[13]. This technique involves calculating the percentage error for agreement between two techniques. This is normally quoted as needing to be less than 30%. This does not mean that the error for the tested technique is 30%, as this is the value of the combination of both standard deviations. In deed if the reference technique has a precision of 10%, a value of 30% would roughly equate to a precision for the mew methodology of 10% also. At one, two and four hours the limits of agreement were 29%, 22% and 28% respectively. At eight hours the percentage error reached 36%. It must be stressed that the pulse pressure algorithm is essentially a software based computer equation, and therefore cannot cause increased errors over time. The increased errors can only be due to one of three causes- one degraded information from the arterial system, for instance from damping of the arterial signal, secondly due to a change in the individual patients haemodynamic profile, specifically changes in arterial compliance, resistance or impedance and lastly due to an error in the reference technique. We are confident in our patients that the arterial signal was of an optimal characteristic, as tested by the square wave test. The errors must therefore be due to either changing haemodynamic characteristics or a higher than expected variability in the lithium dilution cardiac output methodology.

Limitations of our study include the fact that we used a small number of patients in a single centre. Our population had a high level of heterogeneity therefore our results need to be confirmed by bigger studies with more homogeneous groups of patients. This may make our results less generalizable to other populations and settings. We chose to follow the manufacturer's recommendations for the measurement of cardiac output from the lithium dilution technique. This approach utilized only one lithium dilution curve. To our knowledge there is little data describing the inter and intra observer characteristics of performing this technique. To overcome this we ensured that only two researchers who were both extensively trained in the technique performed the measurements. It is of note, that Pittman in his study performed two calibration curves at each time point and in any case of more than 10% variability used an average of three measurements. We did not use this approach, and this may explain some of the variability. It is worth noting, however, that the technique we used is the method recommended by the manufacturers and is how this monitor is used in Intensive Care Units around the world regularly.

## Conclusion

In conclusion we have found that in a mixed group of critically ill patients, the pulse power algorithm remained acceptable for the first four hours following calibration. This finding is valid for the whole group but may mask important changes on individual patients. We thus recommend in the critically ill patient group that the device should be re-calibrated at least every four hours. Until further data becomes available we would suggest re-calibration is performed utilizing at least two lithium dilution curves in order to reduce variability in the technique and improve accuracy. We would also suggest that for any patient where there is a significant change in the haemodynamic status it would be prudent to perform a re-calibration prior to initiating any major therapeutic change even if it is within this four hour window. Further studies should be performed to better understand exactly when re-calibrations should be performed for individual patients, as in some it may be significantly less than the fours hours suggested by this data.

## Competing interests

Dr Rhodes has consulted for Edwards Lifesciences and has performed research for them. Dr Rhodes has lectured for LiDCO.

Dr Cecconi has performed research for Edwards Lifesciences.

## Authors' contributions

MC planned the study, collected the data and performed the statistical analysis, JF collected the data, AR and MG participated in the study design, coordination and in the statistical analysis. All authors read and approved the final manuscript.

## Pre-publication history

The pre-publication history for this paper can be accessed here:


